# The effects of sleep deprivation on cognitive flexibility: a scoping review of outcomes and biological mechanisms

**DOI:** 10.3389/fnins.2025.1626309

**Published:** 2025-07-22

**Authors:** Xuefeng Sun, Zihan Qu, Xiaotu Zhang, Ye Zhang, Xinye Zhang, Haifeng Zhao, Hongshi Zhang

**Affiliations:** ^1^School of Nursing, Chang Chun University of Chinese Medicine, Changchun, China; ^2^Rehabilitation Department, The Second People's Hospital of Dalian, Dalian, China

**Keywords:** sleep deprivation, cognitive flexibility, biological mechanisms, cognitive performance, executive function

## Abstract

**Background:**

Sleep is vital for physical and mental health, yet sleep deprivation is a widespread issue that may impair cognitive flexibility, leading to rigid thinking and slower decision-making. This scoping review synthesizes evidence on the impact of sleep deprivation or sleep loss on cognitive flexibility.

**Objective:**

To provide a comprehensive understanding of the complex and multifaceted effects of sleep deprivation on cognitive flexibility.

**Methods:**

We searched PubMed, Web of Science, ClinicalKey, Cochrane, Scopus, SinoMed, and CNKI for studies evaluating the effects of sleep deprivation on cognitive flexibility. Two researchers independently screened and extracted data, assessing study quality using the Mixed Method Appraisal Tool (MMAT).

**Results:**

Among the 410 retrieved articles, 6 randomized controlled trials and 11 non-randomized studies were included, focusing on the impact of sleep deprivation on cognitive flexibility across children, adolescents, college students, clinicians, athletes, and other adults. Eight studies found that sleep deprivation reduces cognitive flexibility, six reported no significant impact, and two noted temporary improvements due to physical stress. One study highlighted that being overweight exacerbates the negative effects of sleep deprivation on cognitive flexibility.

**Conclusions:**

Sleep deprivation may predominantly impair accuracy rather than reaction time. Total sleep deprivation consistently reduces task-switching accuracy and cognitive flexibility, whereas partial sleep deprivation's effects remain unclear. The primary biological mechanisms involve decreased cerebral oxygen supply, impaired cerebrovascular reactivity, and alterations in gene expression and hormone levels. Rigorous randomized trials with objective measures are needed to assess long-term impacts across populations and age groups.

## Introduction

Sleep can be described as the brain's “valve,” an essential physiological process for the human body (Underwood, 2013). During sleep, the brain opens this “valve,” enabling cerebrospinal fluid to clear metabolic waste, maintain homeostasis, and support memory consolidation and the integration of fragmented daily information (Xie et al., 2013). Upon waking, the brain initiates new neural activities and information processing, thereby enabling normal brain function (Hauglund et al., 2025). However, 25% to 30% of the global population experiences sleep disorders, leading to poor sleep quality or insufficient sleep duration, resulting in sleep deprivation. Sleep deprivation can disrupt the biological clock and neurotransmitter balance, triggering various sleep disorders. These factors interact to form a vicious cycle, collectively impacting both physical and mental health (Kiley et al., 2019).

Sleep deprivation refers to a reduction in both the duration and phases of sleep due to environmental or personal factors, typically defined as obtaining <4 h of sleep within a 24-h period (Khan and Al-Jahdali, 2023). It encompasses both acute and chronic sleep deprivation (Choshen-Hillel et al., 2021). Research indicates that acute, short-term sleep deprivation may merely be a nuisance, but chronic sleep deprivation can have severe consequences for both young and elderly individuals (Madan Jha, 2022). On one hand, sleep deprivation can lead to low mood, irritability, fatigue, forgetfulness, weakened immunity, and varying degrees of memory impairment (Short and Louca, 2015; Stickgold and Walker, 2007). On the other hand, it may also increase the risk of cardiovascular diseases, diabetes, obesity (Tobaldini et al., 2017), and even elevate the likelihood of developing cancer (Huang et al., 2021). Therefore, the National Sleep Foundation (NSF) recommends that young adults aged 18 to 25 should sleep at least 7 to 9 h per day, while older adults aged 65 and above are advised to sleep at least 7 to 8 h daily (Hirshkowitz et al., 2015).

A growing body of research indicates a strong link between sleep deprivation and cognitive decline (Guarnieri, 2019; Jee et al., 2020). Total sleep deprivation exceeding 24 h significantly impairs performance in executive functions (García et al., 2021). Cognitive flexibility, as a core component of executive functions, undergoes significant changes under the influence of sleep deprivation. Given the critical role of cognitive flexibility in adapting to dynamic environments and making quick decisions, it is particularly important in specialized professions such as athletes, clinicians, and astronauts. For individuals in these professions, maintaining a high level of cognitive flexibility is not only a professional requirement but also a critical factor in ensuring workplace safety and efficiency (Whitney et al., 2015). Therefore, research in this area holds significant value. It is noteworthy that most studies have reported connections between sleep deprivation and various cognitive components such as attention, memory, processing speed, and complex reasoning (Hudson et al., 2020; Cousins and Fernández, 2019). However, only a few studies have investigated the impact of sleep deprivation on cognitive flexibility, and the results have been inconsistent (Lim and Dinges, 2010).

Cognitive flexibility refers to the ability to consciously adapt cognitive strategies in response to environmental changes, enabling individuals to adjust to new situations and solve novel problems. At its core, it involves the switching between rules, reflecting an individual's cognitive shifting and inhibitory control abilities (Zühlsdorff et al., 2023). Currently, the Wisconsin Card Sorting Test (WCST) and task-switching paradigms are commonly used to measure cognitive flexibility (Nyhus and Barceló, 2009; Schmitter-Edgecombe and Langill, 2006). However, these methods primarily rely on behavioral data analysis, making it difficult to explore the underlying neural mechanisms. There is limited research utilizing objective techniques, such as electroencephalography (EEG), to investigate cognitive flexibility under conditions of sleep deprivation. Previous studies have reported that the prefrontal cortex plays a critical role in cognitive flexibility. Sleep deprivation reduces blood flow in the prefrontal regions, leading to impaired prefrontal cortex function (Verweij et al., 2014). Sleep deprivation disrupts the balance of glutamate-GABA neurocircuitry in the prefrontal cortex, resulting in altered neuronal firing patterns (Kamal, 2010). Recent research highlights the critical role of brain-derived neurotrophic factor (BDNF) in neuronal development, survival, and synaptic plasticity enhancement. Genetic variations in BDNF may indicate susceptibility to the effects of sleep deprivation (Murer et al., 2001; Bachmann et al., 2012). Research using functional magnetic resonance imaging (fMRI) has found that after sleep deprivation, individuals exhibit significantly reduced activation in the prefrontal cortex when performing cognitive flexibility tasks (Drummond et al., 1999). Additionally, sleep deprivation can trigger oxidative stress, resulting in the accumulation of free radicals that damage brain cells and neural connections, thereby affecting the neural foundations required for cognitive flexibility (Ramanathan and Siegel, 2011). However, there is still no consensus on the relationship between sleep deprivation and cognitive flexibility. For example, a prospective cross-sectional study found that emergency department physicians did not show impaired cognitive flexibility despite experiencing sleep deprivation due to shift work (Persico et al., 2018).

Sleep deprivation, as a potentially significant factor influencing cognitive flexibility, makes it crucial to study the relationship between the two in order to gain a deeper understanding of the underlying mechanisms. Such insights are essential for developing interventions or recommendations aimed at enhancing cognitive flexibility and improving individual performance in learning, work, and daily life. Therefore, this scoping review aims to synthesize existing evidence on the effects of sleep deprivation on cognitive flexibility and explore the relationship between sleep deprivation and cognitive flexibility.

## Materials and methods

The study protocol was registered on the Open Science Framework with the registration number (https://doi.org/10.17605/OSF.IO/YKHBT).

### Research question

The research question encompasses several sub-questions: Firstly, does sleep deprivation affect cognitive flexibility in humans? If so, are there differences in the domains of cognitive flexibility that are impacted? Lastly, does the literature suggest potential mechanisms linking sleep deprivation to impaired cognitive flexibility?

### Literature search

The literature search included articles from the establishment of the databases up to February 28, 2025. These searches were organized and conducted by the librarians. According to the pre-established retrieval strategy, PubMed, Web of Science, ClinicalKey, Cochrane, Scopus, SinoMed, and CNKI databases were performed to identify relevant literature. The main concepts searched included “sleep deprivation” and “cognitive flexibility”. Taking PubMed as an example, the search formula is shown in Table 1.

**Table 1:** Search formula of Pubmed database.

**Pubmed**	
**#1**	((“Sleep Deprivation” [Mesh])) OR (Deprivation, Sleep [Title/Abstract])) OR (REM Sleep Deprivation [Title/Abstract])) OR (Deprivation, REM Sleep[Title/Abstract])) OR (Sleep Deprivation, REM [Title/Abstract])) OR (Sleep Insufficiency [Title/Abstract])) OR (Insufficiencies, Sleep [Title/Abstract])) OR (Insufficiency, Sleep [Title/Abstract])) OR (Sleep Insufficiencies [Title/Abstract])) OR (Insufficient Sleep [Title/Abstract])) OR (Sleep, Insufficient [Title/Abstract])) OR (Inadequate Sleep [Title/Abstract])) OR (Sleep, Inadequate [Title/Abstract])) OR (Sleep Fragmentation [Title/Abstract])) OR (Fragmentation, Sleep [Title/Abstract])) OR (Insufficient Sleep Syndrome [Title/Abstract])) OR (Insufficient Sleep Syndromes [Title/Abstract])) OR (Syndrome, Insufficient Sleep [Title/Abstract])) OR (Sleep Debt [Title/Abstract]))
**#2**	((“Cognitive Flexibility” [Mesh])) OR (Cognitive Flexibilities [Title/Abstract])) OR (Flexibilities, Cognitive [Title/Abstract])) OR (Flexibility, Cognitive [Title/Abstract])) OR (Cognitive Inflexibility [Title/Abstract])) OR (Cognitive Inflexibilities [Title/Abstract])) OR (Inflexibilities, Cognitive [Title/Abstract])) OR (Inflexibility, Cognitive [Title/Abstract]))
**#3**	#1 AND #2

### Inclusion and exclusion criteria

Our study included observational studies with no restrictions on participants' age, gender, race, or other demographic characteristics. These studies were designed to explore the relationship between sleep deprivation and cognitive flexibility. Among them, sleep deprivation could be acute, transient, or chronic. If the literature was only available in the form of an abstract, or was a systematic review in any form, or was written in a language other than English, it would be excluded. Studies assessing the impact of sleep deprivation on other cognitive domains were also not eligible for inclusion. See the Table 2.

**Table 2:** Inclusion and exclusion criteria.

**Inclusion criteria**	**Exclusion criteria**
• Population: Participants of all ages, genders, ethnicity	• The participants exhibited cognitive impairment.
• Exposure factor: Sleep deprivation, and sleep restriction	• Systematic review or meta-analysis, conferences, guidelines, editorials, or commentaries
• Outcomes: Cognitive flexibility, and conversion cost.	• Other types of sleep disorders, such as restless leg syndrome, obstructive sleep apnoea
• Study designs: Observational study (cross sectional, RCT, cohort, surveys)	• Animal experimentation
• English language.	• Non-English

### Study selection

Use Zotero software to manage and screen literature. After removing duplicates, one researcher screened titles and abstracts. Documents describing sleep deprivation as an exposure factor were temporarily retained for full-text review. Another researcher conducted the full-text review, determining inclusion based on predefined criteria. Disagreements were resolved through joint review and consensus.

### Quality appraisal

The Mixed Method Appraisal Tool (MMAT, 2018 version) was used to evaluate the quality of the literature, enabling appropriate weighting and interpretation. The MMAT is designed for systematic reviews and assesses five research categories: qualitative research, randomized controlled trials, quantitative non-randomized studies, quantitative descriptive studies, and mixed methods studies (Hong et al., 2018). Two initial questions must be addressed during evaluation: “S1: Are there clear research questions?” and “S2: Do the collected data address the research questions?” Only if both answers are “yes” can the quality assessment proceed. Each study was evaluated against five criteria, with each met criterion marked by an asterisk (^*^). A study meeting all criteria received five asterisks (^*****^). Two researchers independently scored the studies, resolving discrepancies through discussion. To ensure comprehensiveness, no low-quality studies were excluded (Appendix 1).

### Data extraction

Microsoft Excel was used to manage the collected data. Through group discussions, we created a data extraction form, including fields such as author, publication year, country, study design, participant type, age, sample size, measurement tools for sleep deprivation and cognitive flexibility, and main findings. We also extracted data on potential mechanisms linking sleep deprivation to cognitive flexibility. To clarify this relationship, we categorized and organized the extracted content, highlighting results from individual studies and common patterns across the literature.

## Results

### Search results

**Figure 1:**
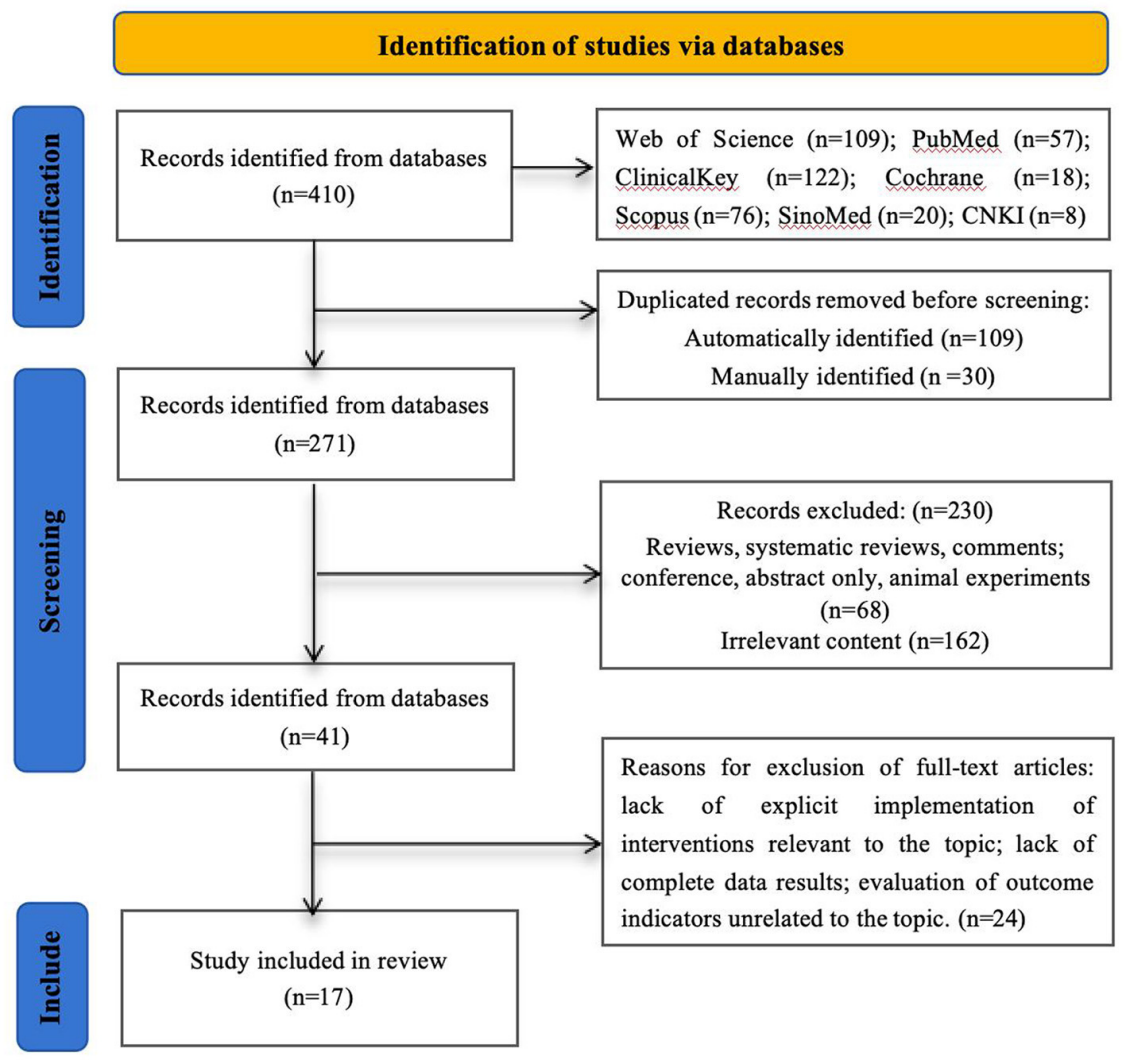
PRISMA 2020 flow diagram.

This review followed the systematic review and meta-analysis (PRISMA 2020) framework (Page et al., 2021). The initial search identified 410 studies. After the automatic and manual removal of duplicate records, 271 studies remained eligible for title and abstract screening. Upon further review of titles and abstracts, systematic reviews, literature reviews, editorials, and similar types of studies were excluded, resulting in 41 studies advancing to the full-text review stage. Ultimately, 17 studies fully met the inclusion criteria (Figure 1). During the data extraction process of these 16 articles, it was discovered that several articles pertained to the same studies but presented outcomes with distinct focal points. During the full-text review stage, the primary reasons for excluding 24 studies included missing critical data and mismatched study contexts.

### Study characteristics

The included articles were published between 1998 and 2024, with 87.5% of them published after 2018. These articles originated from the United States (Randazzo et al., 1998; Whitney et al., 2017; Grant et al., 2018; Honn et al., 2019), France (Maltese et al., 2016; Persico et al., 2018), China (Zhang et al., 2024), Canada (Chan et al., 2024), the United Kingdom (Stager et al., 2024), Ireland (O'Hagan et al., 2018), Egypt (Abdelhamid et al., 2020), Mexico (García et al., 2021), Belgium (Slama et al., 2018), Italy (Ballesio et al., 2018), Turkey (Kiriş, 2022; Sen et al., 2023), and Australia (Pourhassan et al., 2023). Among the 17 studies, six were randomized controlled trials (Randazzo et al., 1998; Honn et al., 2019; Kiriş, 2022; Zhang et al., 2024; Chan et al., 2024; Stager et al., 2024), while the remaining were quantitative non-randomized studies (Maltese et al., 2016; Whitney et al., 2017; Persico et al., 2018; O'Hagan et al., 2018; Grant et al., 2018; Slama et al., 2018; Ballesio et al., 2018; Abdelhamid et al., 2020; García et al., 2021; Sen et al., 2023; Pourhassan et al., 2023). It is evident that the sample sizes of these studies varied (ranging from *n* = 7 to *n* = 66), with the included studies generally featuring small sample sizes, which may be influenced by factors such as study duration, exposure variables, and ethical considerations. The average age of participants ranged from 11.6 to 38.2 years. The study participants included children, adolescents, undergraduate, clinical physicians (anesthesiology residents and emergency department doctors), football players, pilots, and other adults (Table 3).

**Table 3:** Characteristics and analytics of included studies (*N* = 17).

**Author(s) year, country**	**Study design**	**Subjects, sample size**	**Age (years)**	**Procedure of SD**	**Measure of SD**	**Measure of CF**	**Relationship between SD and CF**	**Other cognitive component**
Honn et al. (2019) (USA)	RCT	Adults (*n* = 19) CG: 8 TSD: 11	26.2 ± 5.1	Finished a 4 day/3 night testing CG: 10 h sleep per night TSD: Continuously awake 38 h	N/A	Three-phase reversal learning decision task	Decreased CF (F = 4.61, *P* = 0.032)	N/A
Randazzo et al. (1998) (USA)	RCT	Children (*n* = 16) CG: 8 SD: 8	CG: 12.3 SD: 11.6	Finished a 24-h testing CG: 11 h sleep opportunity SD: 5 h sleep opportunity	PSG	TTCT	No change in CF CG: 94.9 ± 24.0 ms SD: 78.3 ± 11.1 ms (*t* = 1.780, *P* = 0.106)	Language fluency↓
Zhang et al. (2024) (China)	RCT	Undergraduate (*n* = 66) CG: 32 SD: 34	20.46 ± 2.39	Finished one night testing CG: A night of habitual sleep TSD: Continuously awake 24 h	N/A	EEG, Task Switching paradigm	Decreased CF P2 component: (F = 4.430, *P* = 0.039 N2 component: F_(1, 64)_ = 6.866, *P* = 0.011 P3 component: F = 5.549, *P* = 0.022 Time-frequency results: Delta 0.5 s: F = 4.306, *P* = 0.042 Delta 0.7 s: F = 5.584, *P* = 0.021	N/A
Chan et al. (2024) (Canada)	RCT	Adolescents (*n* = 27) CG: 12 SD: 15	CG: 17.1 ± 0.7 SD: 16.7 ± 0.7	Both groups had to complete two pre-set sleep conditions in succession, followed 2-day washout period ①9 h in bed for 5 nights ②6 h in bed for 5 nights	Wrist actigraph Sleep diary	MRI DCCS Test	Decreased CF CG: 56.6 ± 11.1 SD: 50.0 ± 13.0 β = −6.6, *P* = 0.008 95%CI: −11.6~–1.7	Temporal occipital cortex's CVR↓ Inhibitory control↓
Stager et al. (2024) (UK)	RCT	Adolescents (*n* = 61) HW: 31 Obesity: 30	HW: 16.1 ± 1.4 Obesity: 17.6 ± 1.7	After a 2-day washout period of adequate sleep, adolescents completed two sleep conditions ①8 h, 54 min ②4 h, 12 min	Wrist actigraph Sleep diary	DCCS Test	Decreased CF Obesity group: CG: 92.8 ± 3.0 SD: 84.8 ± 3.0 (*P < * 0.001) Increased CF HW group: CG: 95.3 ± 2.9; SD: 97.3 ± 2.9 (*P =* 0.02)	Processing speed↓
O'Hagan et al. (2018) (Ireland)	Quantitative nonrandomized	Pilot (*n* = 7)	21 ± 1.0	Finished two 24 h testing ①8 h sleep opportunity ②no sleep opportunity (SD)	N/A	SCWT	Decreased CF 757.45 ± 58.48 ms (F = 6.475, *P* = 0.004)	TMD↑ Fatigue↓
Abdelhamid et al. (2020) (Egypt)	Quantitative nonrandomized	Anesthesia residents (*n* = 50)	26.88 ± 1.04	**TSD:** Finished a 24 h work shift	KSS	TMT	Decreased CF Pre-shift results: TMT-A:38.82 ± 8.46 ms TMT-B:63.16 ± 11.56 ms Post-shift results: TMT-A: 44.86 ± 9.37 ms TMT-B 72.6 ± 12.294 ms (*P < * 0.001)	Alertness↓
Grant et al. (2018) (USA)	Quantitative nonrandomized	Adults (*n* = 30) Val/Met: 12 Val/Val: 18	Val/Met: 23.17 ± 4.22 Val/Val 22.89 ± 5.18	**TSD:** Continuously awake 24 h	PSG	SCWT	Decreased CF Val/Met: 5.76 ± 4.03 ms Val/Val: 3.84 ± 3.02 ms (*P* = 0.03)	Inhibitory control↓
Persico et al. (2018) (French)	Quantitative nonrandomized	Physicians (*n* = 40) H0: 14 H14: 13 H24: 13	31.3 ± 6.8	Finished one night testing H0: a rest night at home H14: 14 h of night shift H24: 24 h of night shift	N/A	WCST	No change in CF H0: 39.1 ± 9.6 ms H14: 37.5 ± 9.1 ms H24: 40.5 ± 10.8 ms (*P >* 0.05)	Processing speed↓ Working memory ↓ Perceptual reasoning↓
Maltese et al. (2016) (French)	Quantitative nonrandomized	Physicians (*n* = 51) NR: 29 NS: 22	NR: 38.2 ± 7.2 NS: 36.3 ± 7.0	Finished two tests separately, at least 7 days NR: a rest night at home NS: 14 h of night shift	N/A	WCST	No change in CF NR: 41.2 ± 1.2 ms NS: 44.2 ± 1.3 ms (*P* = 0.063)	Processing speed↓ Working memory ↓ Perceptual reasoning↓
Whitney et al. (2017) (USA)	Quantitative nonrandomized	Adults (*n* = 49) CG: 15 SD: 34	27.3 ± 4.8	Finished a 4 day/3 night testing CG: 10 h sleep per night SD: a 10 h baseline sleep opportunity, were subsequently kept awake for 38 h, and then had a 10-h recovery sleep opportunity	VAS	AX-CPT-s	Decreased CF F = 32.0, *P < * 0.001	Alertness**→**
García et al. (2021) (Mexico)	Quantitative nonrandomized	Undergraduate (*n* = 23) CG: 11 SD: 12	CG: 18.73 ± 1.62 SD: 18.08 ± 1.16	CG:Free sleep every night for 3 d SD: Free sleep for one night, stay awake for 24 h the next day, and then free sleep again after SD	None	SCWT	No change in CF Correct responses (%) CG:85.68 ± 9.54 SD:74.59 ± 14.63 Reaction time (ms) CG:644.88 ± 66.89 SD:665.41 ± 82.40 (F = 1.90, *P >* 0.05)	Sustained attention↓ Selective attention↓ Alertness↓
Slama et al. (2018) (Belgium)	Quantitative nonrandomized	Adults (*n* = 35) CG: 17 SD: 18	21.94 ± 2.52	Finished one night testing CG: Sleep at least 7 h a night TSD: Continuously awake 24 h	Wrist actigraph KSS	SCWT	Decreased CF F = 6.97, *P* = 0.013	Working memory**→** Alertness↓
Ballesio et al. (2018) (Italy)	Quantitative nonrandomized	Undergraduate (*n* = 32) CG: 16 SD: 16	CG: 24.31 ± 1.89 SD: 23.50 ± 2.19	Finished one night testing CG: A night of habitual sleep SD: 5 h of sleep allowed	Wrist actigraph	Task Switching paradigm	Increased CF CG: 192.23 ± 201.81 ms SD: 98.99 ± 141.16 ms (*P* = 0.027)	Inhibitory control**→**
Sen et al. (2023) (Turkey)	Quantitative nonrandomized	Adults (*n* = 25)	21.7 ± 2.4	Finished two night testing Day 1: a regular night Day 2: following a 24 h SD	KSS	SCWT	Increased CF Day 1: 57.3 ± 11.8 ms Day 2: 52.4 ± 13.3 ms (*P* = 0.036)	Working memory**→**
Pourhassan et al. (2023) (Austrian)	Quantitative nonrandomized	Soccer athletes (*n* = 21)	25 ± 7.0	Obtain data on sleep interruption time in the natural state over 7 days through sleep devices and sleep diaries.	Wrist actigraph Sleep diary	WCST	No change in CF *r* = −0.11, *P* = 0.703	N/A
Kiriş (2022) (Turkey)	RCT	Adolescents (*n* = 18)	19.3 ± 0.8	Finished four consecutive nights of monitored sleep restriction (6–6.5 h/night) and four nights of sleep extension (10–10.5 h/night)	Actigraphy	CANTAB	No change in CF sleep restriction group: 352.6 ± 60.9 ms sleep extension group: 334.4 ± 41.2 ms *P* = 0.11	Spatial working memory↓

**SD**, Sleep deprivation; **CF**, Cognitive flexibility; **N/A**, Not Applicable; **SCWT**, Stroop Color and Word Test; **“↓”**, Reduced or weakened; **“↑”**, Increased or severe; **“ → ”**, No change**; TMD**, total mood disturbance; **TSD**, total sleep deprivation; **CG**, control group; **PSG**, Polysomnography; **TTCT**, Torrance Tests of Creative Thinking; **KSS**, Karolinska Sleepiness Scale; **TMT**, Trail Making Test; **Val/Met, Val/Val**, Two genotypes of brain-derived neurotrophic factor (BDNF); **EEG**, Electroencephalogram; **WCST**, Wisconsin Card Sorting Test; **VAS**, visual analog scale; **AX-CPT-s**, AX-CPT-s performance testing. Assessed flexible attentional control by measuring subjects' ability to accurately identify valid and invalid cue–probe letter pairings; **MRI**, Magnetic resonance imaging; **HW**, Healthy weight; **DCCS**, Dimensional Change Card Sort. It is a part of NIH Toolbox cognitive battery, which was comprised of 7 tests assessing different cognitive domains, including (1) Flanker test for Inhibitory Control and Attention (FICAT), (2) Dimensional Change Card Sorting (DCCS) test for cognitive flexibility, (3) List Sorting test for Working Memory (LSWM), (4) Picture Sequence Memory Test (PSMT) for visual episodic memory, (5) Pattern Comparison test for Processing Speed (PCPST), (6) Picture Vocabulary Test (PVT) for vocabulary comprehension, and (7) Oral Reading Recognition (ORR) test for reading decoding; **CANTAB**, Cambridge Neuropsychological Test Automated Battery.

### Quality of the included studies

The quality of the included studies was generally high (Randazzo et al., 1998; Maltese et al., 2016; Whitney et al., 2017; Persico et al., 2018; O'Hagan et al., 2018; Grant et al., 2018; Slama et al., 2018; Ballesio et al., 2018; Abdelhamid et al., 2020; García et al., 2021; Kiriş, 2022; Sen et al., 2023; Pourhassan et al., 2023; Chan et al., 2024; Stager et al., 2024), with one study rated as moderate quality (Zhang et al., 2024), and one study rated as low quality (Honn et al., 2019) (Appendix 1).

### Summary of findings

#### Methods and measures of sleep deprivation

Five studies employed total sleep deprivation to observe its effects on cognitive flexibility (Grant et al., 2018; Slama et al., 2018; Honn et al., 2019; Abdelhamid et al., 2020; Zhang et al., 2024), seven studies utilized partial sleep deprivation (Randazzo et al., 1998; Maltese et al., 2016; Persico et al., 2018; Ballesio et al., 2018; Pourhassan et al., 2023; Chan et al., 2024; Stager et al., 2024), four studies adopted a form of alternating between regular sleep schedules and total sleep deprivation (Slama et al., 2018; O'Hagan et al., 2018; García et al., 2021; Sen et al., 2023). Another study investigated the effects of sleep restriction vs. sleep extension on adolescents (Kiriş, 2022). Among the included studies, nine utilized monitoring devices or scales to assess the sleep quality of participants under sleep deprivation conditions. Specifically, two studies employed polysomnography (PSG) (Randazzo et al., 1998; Grant et al., 2018), six studies used wrist actigraphy (Slama et al., 2018; Ballesio et al., 2018; Kiriş, 2022; Pourhassan et al., 2023; Chan et al., 2024; Stager et al., 2024), three studies mentioned the use of sleep diary to record sleep conditions (Pourhassan et al., 2023; Chan et al., 2024; Stager et al., 2024), three studies applied the Karolinska Sleepiness Scale (KSS) (Slama et al., 2018; Abdelhamid et al., 2020; Sen et al., 2023), and only one study utilized the Visual Analog Scale (VAS) for sleep assessment (Whitney et al., 2017). The remaining seven studies did not mention any sleep-related evaluation tools (Table 3).

#### Cognitive flexibility outcome measurement

Among these, five studies utilized the Stroop Color and Word Test (SCWT) (O'Hagan et al., 2018; Grant et al., 2018; Slama et al., 2018; García et al., 2021; Sen et al., 2023), three studies employed the Wisconsin Card Sorting Test (WCST) (Maltese et al., 2016; Persico et al., 2018; Pourhassan et al., 2023), two studies adopted the task-switching paradigm (Ballesio et al., 2018; Zhang et al., 2024), and two studies applied the Dimensional Change Card Sort Test (DDCS) (Chan et al., 2024; Stager et al., 2024). The remaining studies respectively utilized the Trail Making Test (TMT) (Abdelhamid et al., 2020), the Torrance Tests of Creative Thinking (TTCT) (Randazzo et al., 1998), the AX-Continuous Performance Task (AX-CPT-s) (Whitney et al., 2017), the Three-phase Reversal Learning Decision Task (Honn et al., 2019), and Cambridge Neuropsychological Test Automated Battery (Kiriş, 2022). It is noteworthy that only two studies employed objective detection techniques such as EEG and MRI (Zhang et al., 2024; Chan et al., 2024) (Table 3).

#### Relationship between sleep deprivation and cognitive flexibility

Among the 17 studies included in this review, all reported an association between sleep deprivation and cognitive flexibility. Based on the relevant data extracted from Table 3, eight studies indicated that sleep deprivation affects cognitive flexibility (Whitney et al., 2017; O'Hagan et al., 2018; Grant et al., 2018; Slama et al., 2018; Honn et al., 2019; Abdelhamid et al., 2020; Zhang et al., 2024; Chan et al., 2024). Participants with longer duration of sleep deprivation exhibited higher inhibitory costs, task-switching costs, and switch error rates, along with prolonged reaction times. Six studies showed that sleep deprivation did not lead to a decline in cognitive flexibility but significantly impaired cognitive processes such as verbal fluency, working memory, alertness, selective attention, processing speed, and perceptual reasoning (Randazzo et al., 1998; Maltese et al., 2016; Persico et al., 2018; García et al., 2021; Kiriş, 2022; Pourhassan et al., 2023). Surprisingly, two studies conducted in Italy and Turkey, respectively, revealed that cognitive flexibility improved to some extent after sleep deprivation (Ballesio et al., 2018; Sen et al., 2023). One study focused on the cognitive flexibility performance of adolescents in different weight groups following sleep deprivation (Stager et al., 2024). The results demonstrated that overweight or obese adolescents scored significantly lower in cognitive flexibility after sleep deprivation compared to the well-rested group, while adolescents with healthy weight showed improved cognitive flexibility scores after sleep deprivation.

#### Potential biological mechanisms underlying impaired cognitive flexibility

**Figure 2:**
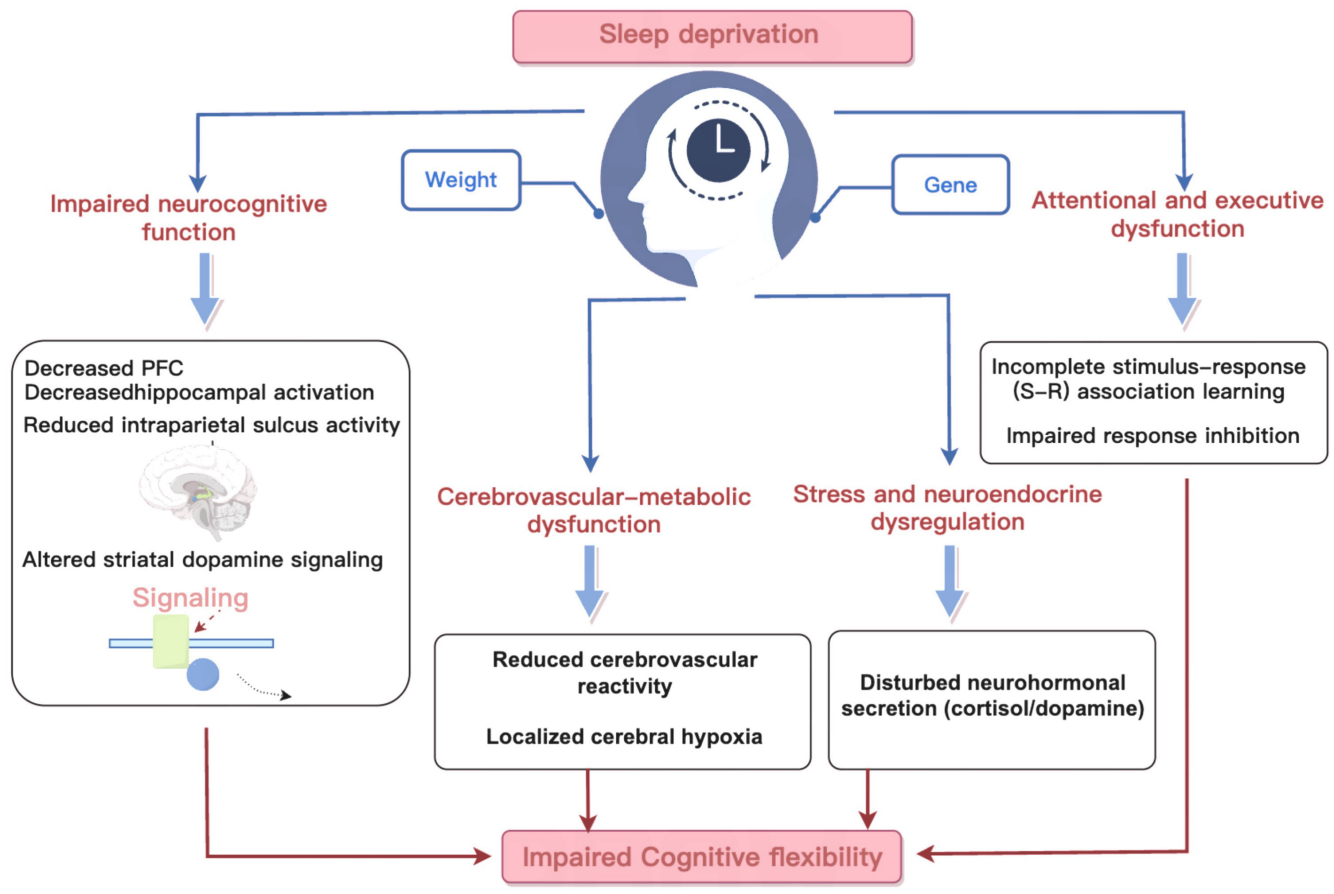
Mechanism diagram of the effects of sleep deprivation on cognitive flexibility.

We also extracted and summarized the mechanisms by which sleep deprivation affects cognitive flexibility as mentioned in the included studies. Eight studies briefly explained the potential mechanisms, which primarily include five types. The first is changes in brain region activity and neurotransmitters: Sleep deprivation reduces insular activity, impairs memory capacity, and affects the expression of brain-derived neurotrophic factor (BDNF). A deficiency in BDNF leads to decreased activity in the prefrontal cortex and hippocampus (O'Hagan et al., 2018; Grant et al., 2018; Honn et al., 2019). The second is related to genes and dopamine: Sleep deprivation affects the binding potential of dopamine D2 receptors (DRD2) (Whitney et al., 2017). The third is the neuroendocrine mechanism: acute sleep deprivation places the body in a state of stress, altering neurotransmitter and hormone secretion (Sen et al., 2023). The fourth is reduced cerebral blood flow: Sleep deprivation decreases cerebrovascular reactivity in specific brain regions, reducing oxygen flow (Chan et al., 2024). Additionally, it may also be associated with overweight and obesity (Stager et al., 2024) (Table 4 and Figure 2).

**Table 4:** Mechanisms of sleep deprivation on cognitive flexibility.

**Author(s), year (country)**	**Subjects**	**Sample size**	**Mechanisms**
Honn et al. (2019)	Adults	19	Sleep deprivation contributes to incomplete or inadequate learning of stimulus-response mappings before reversal.
Zhang et al. (2024)	Undergraduate	66	24 h of sleep deprivation mainly affected the extraction of S-R associations, response inhibition and the allocation of attentional resources, resulting in decreased cognitive flexibility. Further time–frequency analysis found that the ability to suppress interferences and working memory resources may be impaired after 24 h sleep deprivation, which is mainly reflected in the difference between delta band and theta band.
Chan et al. (2024)	Healthy adolescents	27	Sleep deprivation may reduce the cerebrovascular reactivity of specific brain regions (such as the temporo-occipital fusiform cortex and the occipital lobe), decrease the oxygen supply to specific brain regions, and thus affect cognitive flexibility.
Stager et al. (2024)	Adolescents	61	The mechanism by which sleep deprivation affects cognitive flexibility in the brain is related to the degree of obesity. For adolescents who are overweight or obese, sleep deprivation can lead to a decline in their cognitive flexibility, possibly by affecting the physiological functions of the brain. However, adolescents with a normal weight are not affected in this way.
O'Hagan et al. (2018)	Pilot	8	Sleep deprivation leads to reduced activity in the intraparietal sulcus and also affects memory capacity.
Grant et al. (2018)	Adults	30	Sleep deprivation can affect the expression of BDNF, which in turn impacts cortical function. Carriers of the BDNF Met allele have impaired secretion of the BDNF protein. During sleep deprivation, they experience reduced activation in the prefrontal cortex and hippocampus, leading to impaired cognitive flexibility.
Whitney et al. (2017)	Adults	49	The C957T polymorphism of the dopamine D2 receptor gene (DRD2) affects the binding potential of dopamine receptor D2 in the striatum, thereby influencing cognitive flexibility in the brain. There are genetic differences in the effects of sleep deprivation on cognitive function. Individuals with different DRD2 genotypes are affected to varying degrees in cognitive flexibility after sleep deprivation.
Sen et al. (2023)	Adults	25	Short-term acute sleep deprivation can put the body in a stress state, prompting changes in the secretion of neurotransmitters and hormones such as cortisol and dopamine, which in turn affect cognitive flexibility.

## Discussion

While some studies suggest that sleep deprivation does not affect certain executive functions (Binks et al., 1999), cognitive flexibility, as a core component, has been closely linked to sleep deprivation in multiple studies (Stenson et al., 2023). This review highlights extensive research on cognitive flexibility in children, adolescents, and young adults. Honn et al. (2019) found that sleep deprivation reduces sensitivity to neurofeedback, impairing individuals' ability to adjust behavior or decisions based on new information, leading to blunted responses and reduced cognitive flexibility. Three studies consistently demonstrated that sleep deprivation primarily reduces accuracy rather than reaction time, particularly under total sleep deprivation (Slama et al., 2018; García et al., 2021; Zhang et al., 2024). This aligns with prior research indicating that sleep deprivation affects accuracy more than speed (Van Dongen et al., 2003), suggesting that while reaction speed may be maintained, the brain's ability to accurately process information is significantly compromised under sleep deprivation. Similarly, Chan et al. (2024) found that adolescents experienced reduced cognitive flexibility after five nights of sleep restriction. MRI results revealed a significant decrease in cerebrovascular reactivity (CVR) in the fusiform cortex and occipital lobe, suggesting that even short-term sleep restriction may impair cognitive flexibility by affecting cerebrovascular function. Another study utilized EEG to explore the neural mechanisms underlying sleep deprivation's impact on cognitive flexibility in adolescents, employing event-related potentials and time-frequency analysis (Zhang et al., 2024). These studies innovatively applied neuroimaging techniques to objectively examine sleep deprivation's effects on cognitive flexibility at the brain's physiological level, providing valuable insights.

In contrast to previous findings, Ballesio et al. (2018) and Sen et al. (2023) reported increased cognitive flexibility in participants. This may be attributed to short-term, acute sleep deprivation temporarily enhancing alertness and arousal, indirectly boosting cognitive flexibility. However, prolonged sleep deprivation is likely to impair cognitive function. While Randazzo et al. (1998) found no significant impact of sleep deprivation on cognitive flexibility in children, they noted that even one night of sleep restriction could impair abstract thinking, language processing, and other higher-order cognitive functions. A UK study highlighted the role of body weight in adolescents' cognitive flexibility after sleep restriction (Stager et al., 2024). Overweight or obese adolescents exhibited poorer cognitive flexibility and processing speed, while those with a healthy weight remained unaffected, suggesting an interaction between body weight and sleep deprivation in influencing cognitive flexibility.

Second, research on the impact of sleep deprivation on cognitive flexibility in special occupational groups, such as healthcare (Weinger and Ancoli-Israel, 2002) and aerospace (Basner et al., 2014), has gained attention. A survey of pilots found that cognitive flexibility is among the earliest indicators affected by sleep deprivation (O'Hagan et al., 2018). Even short-term sleep deficiency can impair the brain's ability to adapt to new tasks, particularly in high-demand work or study scenarios. Ensuring sufficient sleep is crucial, as it not only affects daily performance but also impacts safety and efficiency in specific occupations. For instance, clinical doctors working night shifts may experience significant cognitive impairments, increasing the risk of misdiagnosis or missed diagnoses. Studies show that sleep deprivation prolongs surgeons' operation times and raises the likelihood of surgical complications (Taffinder et al., 1998). Additionally, physical and mental fatigue is linked to major medical errors, such as syringe confusion and incorrect drug dosages (Barger et al., 2006). Three studies in this review focused on clinical doctors. One found that anesthesiologists experienced significant declines in cognitive flexibility and alertness after 24-h shifts (Abdelhamid et al., 2020). However, the other two studies showed no significant decline in cognitive flexibility after partial sleep deprivation, although processing speed, working memory, and perceptual reasoning were affected (Maltese et al., 2016; Persico et al., 2018). These differences may stem from variations in study populations. For instance, compared to doctors in France, anesthesiologists in Egypt face longer working hours, staff shortages, and greater work pressure, potentially making their cognitive flexibility more vulnerable to sleep deprivation. Similarly, a study of Australian football players found no impact of sleep deprivation on cognitive flexibility (Pourhassan et al., 2023). This may be due to athletes' unique ability to maintain cognitive flexibility under sleep deprivation, possibly through superior compensatory mechanisms. Research suggests that athletes can better allocate attention resources, filter key information, and respond accurately and quickly to cognitive stress caused by sleep deprivation (Souissi et al., 2013).

Furthermore, the review results indicate that the method of sleep deprivation appears to play a significant role in determining the outcomes of cognitive flexibility. Five studies employed total sleep deprivation (Grant et al., 2018; Slama et al., 2018; Honn et al., 2019; Abdelhamid et al., 2020; Hauglund et al., 2025), with participants remaining awake for 24 h, or even up to 38 h, and the results for cognitive flexibility showed a highly consistent trend, all indicating a significant decline. In contrast, studies involving partial sleep deprivation or alternating between normal sleep and total sleep deprivation exhibited inconsistent results in cognitive flexibility tests. This phenomenon suggests that prolonged total sleep deprivation severely impacts the brain's cognitive functions, ultimately leading to a deterioration in cognitive flexibility. However, under conditions of partial sleep deprivation or alternating sleep states, the brain may attempt to maintain cognitive flexibility by reallocating neural resources and enhancing activity in certain key brain regions (Kim et al., 2001; Kiriş, 2022). Nevertheless, partial sleep deprivation could potentially have long-term adverse effects on cognitive flexibility, which warrants further investigation. This also underscores the indispensable role of sleep in maintaining normal cognitive functions of the brain, highlighting that adequate sleep is fundamental to ensuring optimal cognitive flexibility (Raven et al., 2018).

Sleep deprivation has varying effects on cognitive functions at different developmental stages due to differences in brain maturation, physiological characteristics, and compensatory capacities (Sullan et al., 2021; Yang et al., 2022). In childhood, short-term sleep deprivation may not directly impair cognitive flexibility but can disrupt neurodevelopmental processes and compromise higher-order cognitive functions like language processing (Randazzo et al., 1998). Adolescence is a critical period for neural reorganization, particularly in prefrontal-limbic connectivity, influencing cognitive flexibility through the interplay of sleep, cognition, and emotion (Lo et al., 2016). Despite the relatively mature adult brain, sleep deprivation can still impact cognitive flexibility through diverse neural mechanisms. The impact of partial sleep deprivation differs across occupations (Abdelhamid et al., 2020), while total sleep deprivation may result in a “resource depletion” pattern in the brain.

This study also investigated sleep deprivation's effects on other cognitive components, including working memory, alertness, selective attention, sustained attention, inhibitory control, and processing speed. Sleep deprivation typically leads to declines in these functions. Cognitive flexibility depends on the coordination of these processes (Dajani and Uddin, 2015); for example, working memory manages task-related information, while inhibitory control minimizes distractions and errors (Friedman and Robbins, 2022). Alertness and sustained attention also support focus and adaptability during complex tasks (Bastos et al., 2018). Mechanistically, only two studies explored genetic factors (Whitney et al., 2017; Grant et al., 2018), linking the C957T polymorphism of the dopamine receptor D2 gene and the BDNF Met allele to sleep deprivation-induced cognitive flexibility impairments. BDNF Met allele carriers exhibited greater deficits, with higher error rates in Stroop tasks. However, findings from these two genetic loci are insufficient to fully explain the underlying mechanisms.

This review offers valuable insights into the effects of sleep deprivation on cognitive flexibility but is subject to several limitations. The studies included in the review utilized a variety of measurement tools, posing challenges in drawing definitive conclusions despite reporting statistically significant findings. Small sample sizes may restrict the generalizability of results, with most studies relying on task-based assessments and only two incorporating objective detection methods, potentially introducing bias. Moreover, the overrepresentation of young participants warrants caution in extrapolating findings to other age demographics. The brief duration of sleep deprivation experiments may not fully capture the dynamic alterations in cognitive flexibility, resulting in incomplete data. Notably, there is a lack of comparative studies examining partial vs. total sleep deprivation effects on cognitive flexibility. Future investigations should aim to increase sample sizes, prolong study durations, and employ more objective methodologies, such as functional near-infrared spectroscopy, to elucidate the neural mechanisms underpinning the impact of sleep deprivation on cognitive flexibility. Additionally, cognitive flexibility is a multifaceted process influenced by various brain regions, emotional states, and stress levels. Inadequate control of these confounding variables may compromise the accuracy and reliability of study outcomes. In essence, further comprehensive and methodologically rigorous research is imperative to explore the ramifications of sleep deprivation on cognitive flexibility, its underlying mechanisms, and strategies to ameliorate adverse effects and optimize performance in conditions of inadequate sleep.

## Conclusion

These studies reveal that the impact of sleep deprivation on cognitive flexibility varies depending on factors such as demographics, occupations, and experimental setups. While most studies show that sleep deprivation hampers task-switching accuracy and cognitive flexibility, especially evident under total sleep deprivation conditions with consistent outcomes, the effects of partial sleep deprivation on cognitive flexibility and its lasting consequences remain uncertain. The review underscores the necessity for more precise assessment tools, extended study durations, larger participant pools, and longitudinal investigations to elucidate the link between sleep deprivation and cognitive flexibility. The confirmation of the adverse effects of sleep deprivation on cognitive flexibility underscores the potential value of personalized interventions, such as educating individuals on sleep hygiene and providing cognitive training. Prioritizing interventions for adolescents and high-risk professional groups is essential. Implementing a comprehensive system integrating monitoring, intervention, and assessment could effectively enhance cognitive performance, ultimately enhancing overall quality of life.
