# Supplementary material for: The effects of sleep deprivation on cognitive flexibility: a scoping review of outcomes and biological mechanisms
Source: Front Neurosci. 2025 Jul 22;19:1626309. doi: 10.3389/fnins.2025.1626309 (PMC12321868; doi:10.3389/fnins.2025.1626309)
Supplement: Supplementary file 1 [file Data_Sheet_1.docx]

Appendix 1 MMAT quality assessment tool

| **Category of study designs** | **Methodological quality criteria** | Study | | | | | |
| --- | --- | --- | --- | --- | --- | --- | --- |
|  |  | Honn et al.  2019 | Randazzo et al  1998 | Zhang et al.  2024 | Chan et al.  2024 | Stager et al.  2024 | Kiriş.  2022 |
| Screening questions  (for all types) | S1. Are there clear research questions? | √ | √ | √ | √ | √ | √ |
|  | S2. Do the collected data allow to address the research questions? | √ | √ | √ | √ | √ | √ |
|  | *Further* *appraisal* *may* *not* *befeasible* *or* *appropriate* *when* *the* *answer* *is* *‘No’* *or* *‘Can’t* *tell’* *to* *one* *or* *both* *screening* *questions.* | |  |  |  |  |  |
| **2.Quantitative randomized controlled trials** | 2.1. Is randomization appropriately performed? |  | √ |  | √ | √ | √ |
|  | 2.2. Are the groups comparable at baseline? |  | √ | √ | √ | √ | √ |
|  | 2.3. Are there complete outcome data? | √ | √ | √ | √ | √ | √ |
|  | 2.4. Are outcome assessors blinded to the intervention provided? |  |  |  |  |  |  |
|  | 2.5 Did the participants adhere to the assigned intervention? | √ | √ | √ | √ | √ | √ |
|  | Quality evaluation results | ** | **** | *** | **** | **** | **** |

| **Category of study designs** | **Methodological quality criteria** | Study | | | | | |
| --- | --- | --- | --- | --- | --- | --- | --- |
|  |  | O'Hagan et al.  2018 | Abdelhamid et al.  2020 | Grant et al.  2020 | Persico et al.  2018 | Maltese et al.  2016 | Whitney et al.  2023 |
| Screening questions  (for all types) | S1. Are there clear research questions? | √ | √ | √ | √ | √ | √ |
|  | S2. Do the collected data allow to address the research questions? | √ | √ | √ | √ | √ | √ |
|  | *Further* *appraisal* *may* *not* *befeasible* *or* *appropriate* *when* *the* *answer*  *is* *‘No’* *or* *‘Can’t* *tell’* *to* *one* *or* *both* *screening* *questions.* | |  |  |  |  |  |
| **3. Quantitative nonrandomized** | 3.1. Are the participants representative of the target population? | √ | √ | √ | √ | √ | √ |
|  | 3.2. Are measurements appropriate regarding both the outcome and intervention (or exposure)? | √ | √ | √ | √ | √ | √ |
|  | 3.3. Are there complete outcome data? | √ | √ | √ | √ | √ | √ |
|  | 3.4. Are the confounders accounted for in the design and analysis? |  |  |  | √ |  |  |
|  | 3.5. During the study period, is the intervention administered (or exposure occurred) as intended? | √ | √ | √ | √ | √ | √ |
|  | Quality evaluation results | **** | **** | **** | ***** | **** | **** |

continued

| **Category of study designs** | **Methodological quality criteria** | Study | | | | |
| --- | --- | --- | --- | --- | --- | --- |
|  |  | García et al.  2021 | Slama et al.  2018 | Ballesio et al.  2018 | Şen  2023 | Pourhassan et al  2023 |
| Screening questions  (for all types) | S1. Are there clear research questions? | √ | √ | √ | √ | √ |
|  | S2. Do the collected data allow to address the research questions? | √ | √ | √ | √ | √ |
|  | *Further* *appraisal* *may* *not* *befeasible* *or* *appropriate* *when* *the* *answer*  *is* *‘No’* *or* *‘Can’t* *tell’* *to* *one* *or* *both* *screening* *questions.* | |  |  |  |  |
| **3. Quantitative nonrandomized** | 3.1. Are the participants representative of the target population? | √ | √ | √ | √ | √ |
|  | 3.2. Are measurements appropriate regarding both the outcome and intervention (or exposure)? | √ | √ | √ | √ | √ |
|  | 3.3. Are there complete outcome data? | √ | √ | √ | √ | √ |
|  | 3.4. Are the confounders accounted for in the design and analysis? | √ | √ | √ | √ | √ |
|  | 3.5. During the study period, is the intervention administered (or exposure occurred) as intended? | √ | √ | √ | √ |  |
|  | Quality evaluation results | ***** | ***** | ***** | ***** | **** |
